# Insecticidal Activity of 11 Bt toxins and 3 Transgenic Maize Events Expressing Vip3Aa19 to Black Cutworm, *Agrotis ipsilon* (Hufnagel)

**DOI:** 10.3390/insects11040208

**Published:** 2020-03-27

**Authors:** Xiaorui Yan, Junjiao Lu, Meifeng Ren, Yin He, Yueqin Wang, Zhenying Wang, Kanglai He

**Affiliations:** 1 State Key Laboratory for Biology of Plant Diseases and Insect Pests, Institute of Plant Protection, Chinese Academy of Agricultural Sciences, Beijing 100193, China; yanxr1120@126.com (X.Y.); zywang@ippcaas.cn (Z.W.); 2 Institute of Plant Protection, Shanxi Academy of Agricultural Sciences, Taiyuan 030031, Shanxi, China; lujunjiao@126.com (J.L.); sxzbsrmf@163.com (M.R.); 3 Department of Physical Medicine and Rehabilitation, University of Alabama, Birmingham, AL 35401, USA; yhe@uab.edu

**Keywords:** *Agrotis ipsilon*, *Bacillus thuringiensis* toxins, transgenic maize events, resistance

## Abstract

Black cutworm (BCW), *Agrotis ipsilon* (Hufnagel), is an occasional pest of maize that can cause considerable economic loss and injury to corn seedlings. This research mainly assessed the susceptibility of BCW neonates to 11 Bt toxins (Cry1Ab, Cry1Ac, Cry1Ah, Cry1F, Cry1Ie, Cry1B, Cry2Aa, Vip3_ch1, Vip3_ch4, Vip3Ca2, Vip3Aa19) by exposing neonates to an artificial diet containing Bt toxins and evaluated the efficacy of three transgenic maize events (C008, C009, C010) expressing Vip3Aa19 toxin against BCW. The toxin-diet bioassay data indicated that Vip3Aa19 protein (LC_50_ = 0.43 μg/g) was the most active against BCW. Chimeric protein Vip3_ch1 (LC_50_ = 5.53 μg/g), Cry1F (LC_50_ = 83.62 μg/g) and Cry1Ac (LC_50_ = 184.77 μg/g) were less toxic. BCW was very tolerant to the other Bt toxins tested, with LC_50_ values more than 200 μg/g. Greenhouse studies were conducted with artificial infestations at the whorl stage by placing second-instar BCW larvae into whorl leaf and the fourth-instar larvae at the base of maize seedings. These results suggest that these transgenic maize events expressing Vip3Aa19 can provide effective control for BCW.

## 1. Introduction

Black cutworm (BCW), *Agrotis ipsilon* (Hufnagel) (Lepidoptera: Noctuidae), is an occasional pest of maize. Young *A. ipsilon* larvae initially feed on the leaves at the whorl stage, leaving holes on the surface of leaves. As BCW larvae feed, they cut stems at or below the growing point and cause seedlings to die, reducing plant populations and crop yield and causing considerable economic loss [1]. *A. ipsilon* was first recorded in Britain in 1831 and was regarded as native. During 109 years (1831–1939) of flight records, *A. ipsilon* was observed in the British Isles in 64 years [2]. It was also found in Hawaii, Europe, Australia, New Zealand, North Africa, the Pacific Rim and Asia [3,4].

*Bacillus thuringiensis* (Bt) is a Gram-positive bacterium that produces insecticidal Cry (crystal) proteins and Vips (vegetative insecticidal proteins) which are active against insects and other invertebrates [5,6]. Their protoxins are hydrolyzed into active toxins by midgut proteases after ingestion by target pests. The active toxins lyse midgut epithelial cells by inserting into the target membrane and forming pores, which eventually lead to the death of pests [7,8,9]. Although Bt toxins show a high efficacy against some pests, each has its own insecticidal spectrum of toxicity. Only a few studies have evaluated the susceptibility of BCW to Bt toxins. For example, the lethal effect of Cry2Ab is better than that of Cry1Ac against *A. ipsilon* neonate [10], and Cry1Fa2 toxin, isolated from Bt subspecies *aizawai*, has shown some activity against BCW larvae in laboratory studies and in the field [11]. Besides, Yu et al (1997) discovered that after feeding on an artificial diet containing 40 ng/cm^2^ Vip3A protein for 72 h, the midgut epithelial cells were broken, thus leading to the death of larva [12]. 

Transgenic maize producing Bt toxins from soil bacterium Bt provides an effective alternative tool to conventional chemical insecticides for the control of major coleopteran and lepidopteran insect pests, with little adverse effect to beneficial insects and other non-target organisms [13,14,15]. Several laboratory studies and greenhouse trials have shown that the currently developed Bt maize lines were highly toxic to BCW, and event TC1507 expressing the Cry1F protein has made it possible to control a broader spectrum of lepidopteran pests including BCW [16]. Syngenta developed transgenic cotton VipCot^TM^ expressing *vip3Aa19* gene, which is highly toxic to BCW [17]. However, the efficacy of Bt transgenic maize events against BCW, developed by public-sector scientists in China, has not been widely studied. 

The primary objective in the present study was to test the toxicity of 11 Bt toxins (Cry1Ab, Cry1Ac, Cry1Ah, Cry1F, Cry1Ie, Cry1B, Cry2Aa, Vip3_ch1, Vip3_ch4, Vip3Ca2, Vip3Aa19) against BCW in achieving a broader spectrum for pest control and inhibiting resistance. Besides, to prove the practical application effect of Bt transgenic corn in the field, the resistance of transgenic maize with events C008, C009, C010 expressing Vip3Aa19 against BCW was evaluated in greenhouses.

## 2. Materials and Methods

### 2.1. Insects

A lab colony of BCW was used for diet bioassay and plant infestation in a greenhouse. This colony originated from a field collection in Shanxi Province, China, 2013 and was reared on an artificial diet (Patent No. ZL200810112399.7) in the laboratory for 55 generations without exposure to Bt toxins [18].

### 2.2. Bt Toxins

Trypsin-activated Cry1Ab, Cry1Ac and Cry1F (98% pure protein), were produced by Marianne P. Carey, Case Western Reserve University, USA. Cry1Ie and Cry1Ah were chromatographically purified recombinant proteins expressed in *Escherichia coli* and engineered *Bt* acrystalliferous mutant HD73, respectively. Cry1B and Cry2Aa were extracted using the isoelectric point precipitation method. Vip3Aa19 was obtained by IPTG (Isopropyl-beta-D-thiogalactopyranoside) induction and affinity column chromatography (GE Healthcare). Vip3_ch1 and Vip3_ch4, chimeric proteins from Vip3Aa45 and Vip3Ca2, were generated by overlap PCR (Polymerase Chain Reaction) [19]. Vip3Ca2, Vip3_ch1 and Vip3_ch4 were provided by J. Gomis-Cebolla and J. Ferré from the Institute of Biotechnology and Biomedicine, Universitat de València, Spain. All toxins were stored at −80 °C.

### 2.3. Plant Materials

Three transgenic maize events (C008, C009 and C010) developed by Beijing DBN Biotechnology Company, Ltd. expressing Vip3Aa19 toxin and their non-Bt counterpart cultivar as the control (CK) were evaluated in this study. The experiments were conducted separately at the National Agricultural Science and Technology Park (NASTP), Changping, Beijing and at Shanxi Academy of Agricultural Sciences (SAAS) facilities in Taiyuan, Shanxi Province. At NASTP, plants of each event and its non-Bt counterpart control were initially planted in flower pots (L6 cm × W6 cm × H10 cm) with a single seed per pot in the greenhouse. The soil used for planting was KLASMANN 422 (Klasmann-deilmann, Bremerhaven, Germany). After 8 days, the plants were transplanted to a seedling box (L30 cm × W45 cm × H60 cm), which was adapted for infestation with BCW larvae. Each box was randomly transplanted with eight plants of same event or control. In total, there were 23 to 24 transplanted boxes for each event or control. These plants were used for infestation with BCW larvae 24 h after transplanting. At SAAS, plants of each event or its non-Bt counterpart control were randomly planted in six seedling boxes (L35 cm × W24 cm × H12 cm) with 30 seeds per box. These plants were then used for infestation with BCW larvae 8 days after planting.

### 2.4. Diet Bioassays

The toxicity of Bt toxins against BCW was determined in diet bioassays by exposing randomly selected neonate larvae (within 12 h after hatching) to a series of eight to ten concentrations of purified toxin (Table 1) and a distilled water control incorporated into an artificial diet [18] based on wheat germ in 24-well plastic trays, with two plates per toxin concentration or control. Single neonates were picked up with a fine brush (Penicilli size L0.9 cm × W0.15 cm, Maries, Shanghai, China) and transferred to individual wells in 24-well plastic trays to give 48 neonates per test concentration. The trays were covered with membrane (Cat# 3M-9733, Minnesota Mining and Manufacturing Company, Minnesota, USA) and placed in a controlled environment rooms at 26 ± 1 °C and 80% RH under a 16 h light: 8 h dark photoperiod for 7 days. Those larvae that were visibly motionless when poked with a pin were considered to be dead larvae. 

### 2.5. Plant Evaluation

The efficacy of transgenic maize plants in controlling BCW was evaluated with the artificial infestation of the plants in the seedling boxes in greenhouse at the V2–V3 stages. Two experiments were conducted.

Experiment 1: At NASTP, each seedling box was a replicate, and there were 12 boxes for Bt maize event and 11 replicates for control at NASTP (at SAAS, three boxes/replicates for each event and/or control). Plants in a seedling box were artificially infested with second-instar larvae of BCW. Each plant was infested with two larvae (one larva per plant at SAAS) by placing the larvae into the whorl of the plant stage using a fine paint brush. Plant-cutting damage was recorded by counting the number of plants cut at 5, 7 and 11 days after infestation, respectively [20]. 

Experiment 2: At NASTP, each seedling box was a replicate, and there were six boxes for the Bt maize event and six replicates for the control (at SAAS, three boxes/replicates for each event and/or control). Plants in the seedling box were artificially infested with fourth-instar larvae of BCW. Each seedling box was infested with three larvae (at SAAS, 10 larvae per box) by placing a larva at the base of a plant randomly. Plant damage was recorded at 1, 3, 5 and 7 days after infestation, respectively.

The temperature in the greenhouse ranged from 20 °C (night) to 28 °C (day) during the experiment.

### 2.6. Statistical Analysis

Response (mortality)-concentration data from the experiments of diet bioassays were subjected to probit analysis. PoLoPlus V 1.0 (LeOra Software Company, Petaluma, CA, USA) was employed to analyze the susceptibility of BCW to different Bt toxins to generate median lethal concentrations (LC_50_) values with 95% fiducial limits (FL), Chi-Squared (χ^2^), slope with standard errors (slope ± SE). We considered two LC_50_ values to be significantly different only if their 95% fiducial limits did not overlap.

The effects of protecting against BCW damage were determined by the maintained seedling rate (y), which was calculated as $y\left(\%\right)=\frac{n-n_{d}}{n}\times 100$, where *n* and *n*_d_ are the number of infested plants in a box (total plants per box) and the number of cut-down plants, respectively. Data were analyzed by using the PROC GLM (General Linear Models) Procedure (SAS 9.4 software, SAS Institute, North Carolina State University, Raleigh, NC, USA) for repeated measures analysis of variance, with time (damage recording days) as the repeated measure term and cross locations (NASTP and SAAS) to compare the difference of maintained seedling rates between Bt maize events and their negative controls. Percentage data (maintained seedling rates) were transformed with arcsine transformation to ensure the normality of the dataset to be used for statistical analysis. 

## 3. Results

### 3.1. Susceptibility of BCW to Bt Toxins

Bioassay results of Cry and Vip3 toxins against *A. ipsilon* are shown in Table 2. The toxicity of Vip3Aa19 was significantly higher compared to other Bt toxins as there was a non-overlap of 95% fiducial limits between Vip3Aa19 and other Bt toxins. Vip3_ch1 was less toxic than Vip3Aa19, followed by Cry1F and Cry1Ac. For the other tested toxins (Cry1Ab, Cry1B, Cry2Aa, Vip3Ca2, Cry1Ah, Vip3_ch4, Cry1Ie), the highest concentrations tested did not kill 50% of the larvae, and we could not accurately estimate the LC_50_ values; all the LC_50_ values were more than 200 μg/g, indicating that these toxins have less susceptibility to BCW.

### 3.2. Efficacy of Plant Resistance

#### 3.2.1. Against Second-Instar Larvae

**Figure 1:**
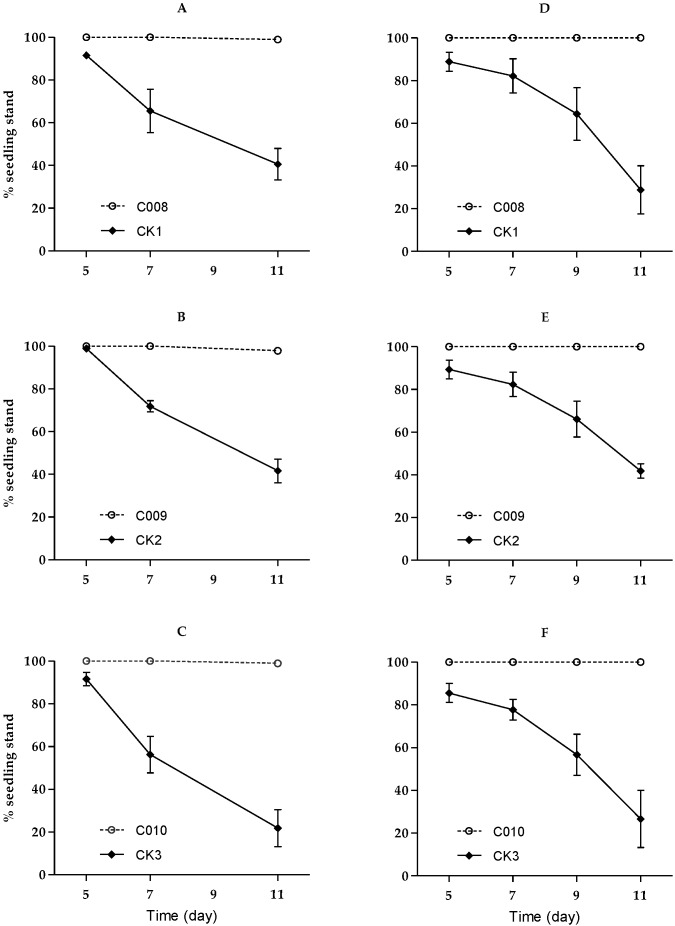
Percentage of seedlings standing of Bt transgenic maize events (C008, C009 and C010) and CK (control) under infestation of second-instar larvae of BCW (at National Agricultural Science and Technology Park (NASTP): (**A**–**C**), and at Shanxi Academy of Agricultural Sciences (SAAS) (**D**–**F**); bar lines: ±SE).

The second-instar larvae bored and damaged whorl leaves 1 to 4 days after infestation. There were few further damages observed on plants in Bt maize seedling boxes on days 5 to 11 after infestation, and there was only one cut on day 11 in Event C008 and Event C010 seedling boxes and two in Event C009 seedling boxes at NASTP trials; no plant cut down had been observed on day 11 in three events at SAAS trials. In contrast, plant cut down was found in the control seedling boxes on day 5 after infestation, and the number of plants standing was significantly decreased over time in both trials (C008: *F*_2,28_ = 44.69, *p* < 0.0001; C009: *F*_2,28_ = 54.68, *p* < 0.0001; C010: *F*_2.28_ = 38.59, *p* < 0.0001) (Figure 1). The percentage of seedlings standing was significantly higher for the three transgenic maize events expressing Vip3Aa19 protein compared with their non-Bt counterpart control (C008: *F*_1,14_ = 141.81, *p* < 0.0001; C009: *F*_1,14_ = 277.47, *p* < 0.0001; C010: *F*_1,14_ = 115.23, *p* < 0.0001).

#### 3.2.2. Against Fourth-Instar Larvae

**Figure 2:**
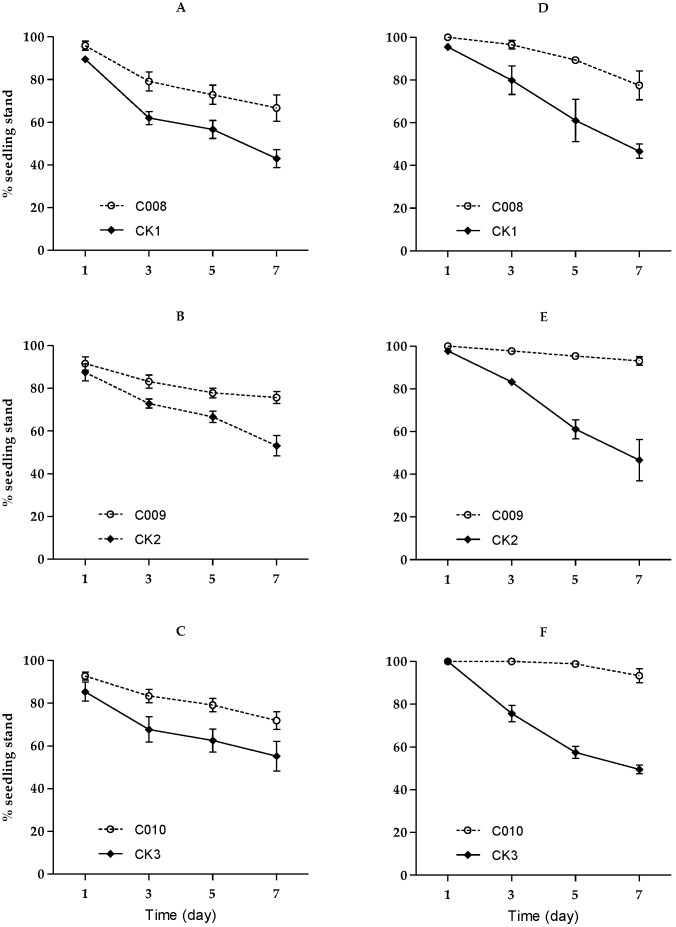
Percentage of seedlings standing of Bt transgenic maize events (C008, C009 and C010) and CK (control) under infestation of fourth-instar larvae of BCW (at NASTP: (**A**–**C**); at SAAS: (**D**–**F**); bar lines: ±SE).

The fourth-instar larvae cut stems near the soil surface level as soon as on the first night after infestation, which caused seedlings to die. The number of corn seedlings uncut significantly decreased over time (C008: *F*_3,42_ = 111.67, *p* < 0.0001; C009: *F*_3,42_ = 42.86, *p* < 0.0001; C010: *F*_3,42_ = 52.22, *p* < 0.0001) (Figure 2). On average, one larva cut 1.4 to 1.6 plants down in non-Bt maize seedling boxes 7 days after infestation, while one larva cut only 0.6 to 0.9 and 0.2-0.8 plants down in Bt maize seedling boxes in NASTP and SAAS trials, respectively. The percentages of seedlings uncut were significantly higher in Bt maize seedling boxes than in their non-Bt counterpart control boxes (C008: *F*_1,14_ = 27.97, *p* = 0.0001; C009: *F*_1,14_ = 32.51, *p* < 0.0001; C010: *F*_1,14_ = 33.11, *p* < 0.0001) In addition, Event C090 and Event C010 performed significantly better in terms of seedling protection in SAAS trials than that NASTP trials (C009: *F*_1,14_ = 9.77, *p* = 0.0074; C010: *F*_1,14_ = 6.66, *p* = 0.0217), but there was no significant difference between the two locations regarding the performance of Event C008 (C008: *F*_1,14_ = 0.92, *p* = 0.3534).

## 4. Discussion

Species-specificity is a distinguished advantage of Bt toxins, which function to kill target insect pests with few or no adverse effects on natural enemies or beneficial and other non-target species. Identifying and selecting toxins that have high toxicity against specific crop target pests is therefore of primary importance in developing insect-resistant Bt crops, which provides the possibility to achieve a “high dose”. Results from this study indicated that BCW was highly tolerant to most of the tested toxins. Of the 11 tested toxins, Vip3Aa19 exhibited about 13-fold (Vip3_ch1) to 1163-fold (Cry1Ie) more toxicity to BCW compared to other Bt toxins. Although Cry1F was the most toxic among all the tested Cry1 toxins, it was still 194-fold less toxic than Vip3Aa19.

Vip3A is well known for its wide spectrum of toxic activity to lepidopteran, especially Nuctuidae insect pests such as *Helicoverpa armigera*, *Spodoptera frugiperda*, *Spodoptera exigua*, but no toxic activity has been shown to *Ostrinia nubilalis* and *Ostrinia furnacalis* [21,22,23]. Vip3Aa has no structural similarity with Cry1 and Cry2 proteins and has a different mode of action [12,24,25]. Vip3Aa is therefore considered to be a good candidate co-expressed with Cry proteins in Bt crops for targeting a broad spectrum and delaying the evolution of insect resistance. A Vip3Aa-selected strain of *Heliothis virescens* that showed more than 2040-fold resistance was as susceptible to Cry1Ac as the unselected control strain, and showed a low level of cross-resistance (seven-fold) to Cry1Ab [26]. On the other hand, the estimated frequency of alleles conferring resistance to Vip3Aa was higher than expected, at 0.034 for *H. armigera* and 0.010 for *H. punctigera*, although Bt crops producing Vip3Aa have not been made commercially available in Australia [27,28].

Cotton containing Cry1Ab stacked with Vip3Aa19 could perform with greater broad-spectrum performance (*S. exigua* and *S. frugiperda*, *Heliothis assulta*, *H. virescens*, as well as *H. armigera*, *Helicoverpa zea*) than either Cry1Ab (higher efficacy against *H. virescens*) or Vip3Aa19 (higher efficacy against *S. frugiperda*. and *H. zea*) alone [29,30,31]. Corns expressing Cry1Ab (event Bt11 and MON810) or Cry1Fa2 (Event TC1507) demonstrate high efficacy against *O. nubilalis* [14,20,32], but they are unlikely to have a significant impact on BCW [1,33]. However, either the Cry1Ab × Vip3Aa20 pyramid of Bt11×MIR162 corn or Cry1Fa2 × Cry1Ab × Vip3Aa20 pyramid of TC150 × MON810 × MIR162 corn could provide a much broader-spectrum high dose against *H. zea* and *S. frugiperda* [34,35,36,37] as well as BCW [16]. In this study, transgenic corn expressing Vip3Aa19 (events C008 and C010) demonstrated high efficacy to the control of BCW, which suggests that it is a good candidate to be stacked with corn targeting *O. furnacalis* and *Mathimna separata* (such as Cry1Ab, Cry1F corn) to obtain a much broader spectrum.

Vip3A is well known and has been used in commercial Bt crops for the last 10 years; however, few studies have reported on Vip3Ca’s insecticidal spectrum [38,39,40]. There are three types of Vip3Ca—i.e., Vip3Ca1, Vip3Ca2 and Vip3Ca3—which are only different at the molecular level, consisting of two point mutations rendering nonsynonymous substitutions [38]. Vip3Ca3 is barely toxic to the European corn borer (*O. nubilalis*) and Asian corn borer (*O. furnacalis,* a relative species of *O. nubilalis*), and has low toxicity to *A. ipsilon* [38]. Vip3Ca2 is slightly toxic to *O. nubilalis* [39]; however, it is highly toxic to both susceptible and resistant *O. furnacalis* [24]. In the present study, marginal toxic activity was observed for *A. ipsilon* with Vip3Ca2 (mortality = 33.3% at a dose of 200 μg/g).

A previous report documents that Vip3AcAa, a chimeric protein combined with the N-terminal part of Vip3Ac and the C-terminal part of Vip3Aa, has a broad spectrum against *Spodoptera litura*, *S. exigua* and *A. ipsilon* [41]. Vip3_ch1 is a chimeric protein with the N-terminal part of Vip3Ca2; i.e., domain I, as proposed for the Vip3Af domains by Quan and Ferré (2019), and the rest of the domains are targeted by Vip3Aa45 (domains II–V, as proposed for the Vip3Af domains by Quan and Ferré (2019)) [42], which is expected to have additional potential for the control of lepidopteran pests. In this study, Vip3_ch1 demonstrated a significant increase in insecticidal activity against BCW compared to Vip3Ca2, which was shown to be almost nontoxic (Table 1). The lethal dose (LC_50_) ratio of Vip3_ch1 was 15-fold higher than Cry1F, but it was 12-fold lower than Vip3Aa19. Similar to Vip3Aa16 [24], Vip3_ch1 was nontoxic to *O. furnacalis* [19]. 

Vip3_ch4 is a chimeric protein with a central domain; i.e., domains II and III, as proposed for the Vip3Af domains by Quan and Ferré (2019) [41] from Vip3Aa45, and domain I as well as domains IV and V from Vip3Ca2. It demonstrates insecticidal activity against *O. furnacalis*, but does not show toxicity to *S. frugiperda, H. armigera*, *Anticarsia gemmatalis*, and *Mamestra brassicae* [19]. In this study, Vip3_ch4 was nontoxic to BCW, confirming a loss of toxicity to Noctuidae pests.

The Cry2A toxins have been reported to have highly insecticidal activity against several lepidoptera insects. The larvicidal proteins demonstrate especially high toxicity against some important crop pests of Noctuidae insects, such as *H. armigera* and *H. zea*, and are functional with a different mode of action from Cry1A toxins, which are considered to be significant implications for insect resistance management [43]. Cry2Aa is toxic to the velvetbean caterpillar *A. gemmatalis*, but not toxic to *S. frugiperda* [44]. However, Fiuza et al. (2013) report that Cry2Aa is not toxic to *A. gemmatalis* [45]. Cry2Aa is less toxic (LC_50_ = 4.30 μg/g) to *H. virescens* and *H. armigera* than Cry1Ac (LC_50_ = 0.73 μg/g) with lethal dose ratios of 5.89 and 6.54 of LC_50_, respectively [46,47]. In this study, Cry2Aa exhibited less toxicity to BCW than Cry1Ac and Cry1F.

All Cry1 toxins tested in this study were less toxic than Vip3A toxins to BCW. Among them, Cry1F showed the highest toxic activity, followed by Cry1Ac. The lethal ratios were 195-fold and 430-fold lower than Vip3Aa19 based on the LC_50_s. Cry1Ab, Cry1Ah and Cry1B were marginally toxic, and Cry1Ie was completely nontoxic. This confirms the previous findings that Cry1Ab and Cry1Ac have low toxicity against BCW [10,21,48], which points to BCW as an unsuitable target or even a non-target for these toxins. Furthermore, it can be concluded that Vip3Aa has the greatest toxic effect on BCW, and Bt maize Event C090 and Event C010 can be recommended as means for BCW control.

## 5. Conclusions

Vip3Aa19 showed highly toxic activity to BCW and transgenic Bt maize events expressing Vip3Aa19 can provide effective control for BCW, which could offer a sustainable biological agent option for pest insects management.

## Figures and Tables

**Table 1:** Bt toxin concentrations used for bioassay.

Toxins	Concentration (μg/g, Toxin/Diet)									
Cry1Ab	0	1.0	2.0	5.0	10.0	20.0	50.0	100	200	
Cry1Ac	0	1.0	10.0	50.0	100	200	500	800		
Cry1Ah	0	1.0	2.0	5.0	10.0	20.0	50.0	100	200	300
Cry1F	0	1.0	2.0	5.0	10.0	20.0	50.0	100		
Cry1Ie	0	1.0	2.0	5.0	10.0	20.0	50.0	100	200	500
Cry1B	0	1.0	2.0	5.0	10.0	20.0	50.0	100	200	
Cry2Aa	0	1.0	2.0	5.0	10.0	20.0	50.0	100	200	
Vip3Aa19	0	0.05	0.1	0.2	0.5	1.0	2.0	5.0		
Vip3Ca	0	1.0	2.0	5.0	10.0	20.0	50.0	100	200	
Vip3_ch4	0	1.0	2.0	5.0	10.0	20.0	50.0	100	200	400
Vip3_ch1	0	1.0	2.0	5.0	10.0	20.0	50.0	100	200	400

**Table 2:** Toxicity of 11 Bt toxins against the black cutworm (BCW), *Agrotis ipsilon* (Hufnagel).

**Toxins**	**n**	**LC_50_ (95% FL)** **μg/g**	**LC_90_ (95% FL)** **μg/g**	**Slope ± SE**	**χ^2^**	**df** **(χ^2^)**
Vip3Aa19	384	0.43 (0.35–0.53)	1.96 (1.46–2.89)	1.95 ± 0.17	4.7	12
Vip3_ch1	432	5.53 (4.13–7.23)	50.75 (33.87–89.20)	1.33 ± 0.14	8.0	13
Cry1F	336	83.62 (48.73–203.49)	>1000	0.98 ± 0.15	6.7	11
Cry1Ac	336	184.77 (126.74–282.66)	>4000	0.93 ± 0.11	4.8	11
		**Concentration ^a^** **(μg/g)**	**Mortality ^b^** **(%)**			
Control	432	0	0			
Cry1Ab	384	200	18.7			
Cry1B	384	200	14.6			
Cry2Aa	384	200	50.0			
Vip3Ca2	384	200	37.5			
Cry1Ah	432	300	29.2			
Vip3_ch4	432	400	14.6			
Cry1Ie	432	500	0.0			

n, number of larvae tested; ^a^, the highest concentration tested; ^b^, mortality at the highest concentration.
